# Development and Characterization of Polymorphic Microsatellite Markers for *Sedum sarmentosum* (Crassulaceae) and Their Cross-Species Transferability

**DOI:** 10.3390/molecules201119669

**Published:** 2015-11-05

**Authors:** Jing Xu, Fu-Yuan Hou, Ding-Rong Wan, Sha Wang, Dong-Mei Xu, Guang-Zhong Yang

**Affiliations:** College of Pharmacy, South Central University for Nationalities, 708 Minyuan Road, Wuhan 430074, China; houfuyuany@sina.com (F.-Y.H.); wandr666@163.com (D.-R.W.); shaqueen@sina.com (S.W.); xudongmei777@sina.com (D.-M.X.); yanggz888@126.com (G.-Z.Y.)

**Keywords:** *Sedum sarmentosum*, microsatellite markers, polymorphism, genetic diversity, transferability

## Abstract

*Sedum sarmentosum* is an important Chinese medicinal herb that exhibits anti-inflammatory, anti-angiogenic and anti-nociceptive properties. However, little is known about its genetic background. The first set of 14 microsatellite markers were isolated and characterized for *S. sarmentosum* using an SSR-enriched library. Fourteen polymorphic microsatellite markers were acquired with satisfactory amplifications and a polymorphic pattern in 48 *S. sarmentosum* individuals. The number of alleles ranged from 3 to 15. The observed and expected heterozygosities varied from 0.0833 to 0.8750 and 0.2168 to 0.9063, respectively. Two loci showed significant departure from the Hardy-Weinberg equilibrium. Cross-species amplification was carried out in other *Sedum* species. High rates of cross-species amplification were observed. The transferability value ranged from 85.7% in *S. lineare* to 64.3% in *S. ellacombianum*. These markers will be valuable for studying the genetic variation, population structure and germplasm characterization of *S. sarmentosum* and related *Sedum* species.

## 1. Introduction

The *Sedum* L. genus in the Crassulaceae family consists of 470 species that are distributed in the Northern Hemisphere, Africa and Latin America of Southern Hemisphere [1]. *Sedum* L. species are widely used in folk medicine, especially *Sedum sarmentosum*, which has been frequently used for the treatment of chronic hepatitis in China [2,3]. Furthermore, *S. sarmentosum* exhibits anti-angiogenic, anti-cancer, anti-viral and anti-nociceptive properties [4] and is attracting increasing interest from domestic and foreign pharmaceutical companies. The high demand of *S. sarmentosum* for medicinal uses has led to overexploitation of the wild populations, driving them close to local extinction in the wild [5]. Furthermore, the species of *S. sarmentosum* are difficult to identify because of the large variety and close resemblance in closely related genera. For example, it is usually morphologically confused with *S. lineare* [6]. Plants of the *Sedum* L. genus usually differ in components and activities, and therefore classification and identification of the medicinal plant species is critical to ensure quality and therapeutic efficacy. Accurate evaluation of its genetic diversity is necessary for the conservation and molecular identification of this species.

Earlier morphological and chemical composition studies have been performed for identification and classification of *Sedum* genus [7,8]. Allozyme markers have been used to assess the genetic relationship of two *Sedum* species [9]. However, little is known regarding the diversity and genetic structure of the *Sedum* genus. DNA markers, such as random amplified polymorphic DNA, have been established to study the genetic relationship of six *Sedum* species. However, the RAPD marker had limited ability to provide stable genetic relationship [10]. More reliable molecular markers are needed to enhance genetic analyses of *Sedum* species.

Microsatellite or simple sequence repeat (SSR) markers are considered to be effective in accessing genetic diversity and structure of plant population due to their abundance, high level of polymorphism, co-dominance, bi-parentally inheritance, and reproducibility of the results [11,12]. However, microsatellite markers have not been isolated from any *Sedum* species. In the absence of sequenced genomes, microsatellite-enrichment of genomic libraries has been shown to be effective at developing molecular markers for genetic studies [13,14]. In this study, the first set of genomic polymorphic microsatellite markers of *S. sarmentosum* have been developed using a microsatellite-enriched library and tested for transferability across five other *Sedum* species. The polymorphic microsatellite markers might have important implications for the identification, genetic diversity and population genetic structure of *Sedum* genus.

## 2. Results and Discussion

From the 198 colonies screened for inserts, 82 positive colonies were randomly chosen and 68 colonies were successfully sequenced, 7 colonies were duplicates. Fifty-three microsatellite motifs were discovered in 61 singleton sequences. The enrichment efficiency of microsatellites (86.9%) was higher than for *Rubus coreanus* at 84.5% [15], groundnut at 68% [16], and Japanese apricot at 57.0% [17]. Most of the microsatellite motifs (45, 84.9%) were dinucleotides repeats, followed by trinucleotides repeats (5, 9.4%) and repeat motifs that were tetranucleotide or greater (3, 5.7%). Among the dinucleotides repeats, the GT/TG/AC/CA motifs showed the most frequency (37, 82.2%).

38 fragments containing microsatellite motifs that showed flanking regions were used to design primers (partial results are shown in Table 1) and tested for consistency of amplification and polymorphism with 48 *S. sarmentosum*. After preliminary screening, 14 polymorphic microsatellite loci yielded satisfactory amplifications with a polymorphic pattern. The number of polymorphic microsatellite loci observed with respect to amplified loci in *S. sarmentosum* (36.8%) is higher than that obtained by enriched libraries constructed from other medicinal herb, such as the *Dendrobium huoshanense* (30.6%) [18], *Rhodiola* (25.8%) [19], and *Bletilla striata* (13.2%) [20]. Several factors may account for these isolation efficiency differences, including microsatellite frequency within the genome of the studied species, structure of the microsatellites and their flanking regions [21,22,23].

The genetic diversity parameters of the 14 polymorphic microsatellite loci were calculated for *S. sarmentosum* and summarized in Table 2. The number of alleles per locus ranged from three for locus Ssa10 to 15 for locus Ssa17 with an average of 6.9 alleles/locus. The observed heterozygosity (*H*_O_) ranged from 0.0833 to 0.8750 and the expected heterozygosity (*H*_E_) varied from 0.2168 to 0.9063. After sequential Bonferroni correction, two of the loci (Ssa10 and Ssa 60) showed significant departure from Hardy-Weinberg equilibrium (HWE), because of an excess of homozygotes. Null alleles, inbreeding, Wahlund effect, and small population size might lead to the excess of homozygotes [24,25]. The presence of null alleles at the microsatellite loci will be detected by software (e.g., MICRO-CHECKER [26]) and the effects of sample and habitat size will be determined in the future population genetic studies of *S. sarmentosum*. Among the 14 microsatellite loci, no significant linkage disequilibrium was identified between loci.

**Table 1:** Characteristics of 14 polymorphic microsatellite markers isolated from *S. sarmentosum*.

Locus	Repeat Motif	Primer Sequence (5′-3′)	*T_a_* (°C)	Size (bp)	GenBank Accession No.
Ssa 47	(GT)_5_	F: GGAGAAGAGAGAAGAGAGGATG	55	109	KP742353
		R: CACCGTCAAGTAATTCAGTATAAAT			
Ssa 30	(GT)_7_	F: TGGGTGGATTATTGATGAGG	55	112	KP742354
		R: GCTTCCTACTCAATGCAAAACC			
Ssa 92	(TG)_11_	F: TGGAGTGAGTTTTAGGTTTT	51	70	KP742355
		R: CACTGGAAGTGGTACGATAC			
Ssa 46	(TG)_10_	F: AGTGAGTTTTAGGTTTTTGTGT	51	59	KP742356
		R: AAGTGGTACGATACTATTCGC			
Ssa 64	(TG)_11_	F: AATGGAGTGAGTTTTAGGT	51	70	KP742357
		R: CTGGAAGTGGTACGATAC			
Ssa 17	(CA)_7_	F: TCAGGCTCCATAGTAACCC	57	173	KP742358
		R: AAGTCGTGTCAGGAAGGC			
Ssa 66B	(CA)_11_	F: CTGGAAGTGGTACGATAC	55	67	KP742359
		R: GGAGTGAGTTTTAGGTTTT			
Ssa 56	(CA)_12_	F: TATTTCGATACTTCAATCACAC	51	105	KP742360
		R: TGTTTATTATTGACATTGAATTG			
Ssa 32	(CA)_10_	F: GGAAGGAGGTTTGGTAGAT	51	118	KP742361
		R: TCATCCTGTGACCCCTGT			
Ssa 10	(CA)_7_–(CA)_13_	F: ACTGGAAGTGGTACGATACTATT	52	223	KP742362
		R: GAAATGTGTACTTACCTTATCCA			
Ssa 66A	(GT)_7_	F: GGTTGCATTGCATAGCC	52	234	KP742363
		R: AGAATCTTCTCTCCAGAGTCA			
Ssa 83	(GT)_8_	F: AGGAAGGCGAATGAGTGT	55	153	KP742364
		R: AAGAAGGTGAAATGTATAGCA			
Ssa 60	(TGTTGTG)_6_	F: GCTTCTTGCTGAAAGTGACA	55	243	KP742366
		R: ACGACAGGTTTCCCGACT			
Ssa 90	(TG)_5_	F: AACAACAGGTTATACCACTTCG	54	128	KP742365
		R: CCACACAAACACACGCAC			

Annealing temperature (*T_a_*).

**Table 2:** Genetic diversity of microsatellite marker for *S. sarmentosum* and *S. lineare*.

Locus	*Sedum sarmentosum* (*n* = 48)				*Sedum lineare* (*n* = 12)			
	*A*	*H*_O_	*H*_E_	*P_HWE_*	*A*	*H*_O_	*H*_E_	*P_HWE_*
Ssa 47	6	0.5892	0.5833	0.6073	–			
Ssa 30	5	0.4583	0.5052	0.0612	1			
Ssa 92	8	0.6458	0.6516	0.8734	4	0.2033	0.2964	0.0312
Ssa 46	6	0.5833	0.6038	0.9824	1			
Ssa 64	7	0.6471	0.5882	0.6667	5	0.45	0.5018	0.3506
Ssa 17	15	0.8750	0.9011	0.1823	7	0.5	0.6012	0.2021
Ssa 66B	5	0.4512	0.3958	0.4612	6	0.4583	0.5821	**0.0000**
Ssa 56	10	0.8333	0.9063	0.0672	6	0.4167	0.5623	0.0313
Ssa 32	4	0.4167	0.4920	0.7343	1			
Ssa 10	3	0.0833	0.2168	**0.0000**	1			
Ssa 66A	8	0.5833	0.6328	0.1273	–			
Ssa 83	7	0.5417	0.6027	0.4263	1			
Ssa 60	4	0.2500	0.4328	**0.0003**	1			
Ssa 90	9	0.7033	0.7108	0.6230	3	0.3333	0.3367	0.0421

Number of alleles (*A*), observed heterozygosity (*H*_O_), expected heterozygosity (*H*_E_), and the test for deviation from Hardy-Weinberg Equilibrium (*P*_HWE_). Values in bold represent significant deviation from Hardy-Weinberg equilibrium.

Cross-species amplification tests were performed in the *S. lineare*, which is morphologically close to *S. sarmentosum*. Twelve of the 14 microsatellite markers were amplified successfully and only six showed polymorphism in 12 *S. lineare* individuals (Table 2), which may result from the small size of the populations. The number of alleles per locus ranged from 3 to 7, the *H*_O_ ranged from 0.2033 to 0.5000 and *H*_E_ varied from 0.2964 to 0.6012 (Table 2). Among the six polymorphic microsatellite loci, only one locus (Ssa 66B) deviated significantly from HWE. This result showed that the microsatellite primers could be used in *S. lineare* and selective use of the primers could effectively discriminate *S. sarmentosum* from *S. lineare*.

We also applied the 14 microsatellite markers to test their transferability to other four *Sedum* species (*S. emarginatum*, *S. bulbiferum*, *S. aizoo* and *S. ellacombianum*, Supplementary Table). The tested SSR primer pairs displayed a high amplification frequency across the species. A total of eight (57.1%) markers successfully amplified in all the four other *Sedum* species. The transferability value was highest in *S. emarginatum* (78.6%), followed by *S. bulbiferum* (71.4%) and *S. aizoo* (71.4%) (Table 3). *S. ellacombianum* showed the lowest transferability, only 64.3%. Wu *et al.* used PAPD to study the genetic relationship of six *Sedum* species. The phylogenetic analysis revealed that *S. sarmentosum* and *S. lineare* had the nearest the distance and grouped in one clade, while *S. ellacombianum* was in another clade [10]. The transferability of the SSR markers obtained by enriched libraries was a little lower than that by sequencing, which was expected due to the conservative nature of transcribed regions [27,28,29].

**Table 3:** Transferability of 14 microsatellite markers for the *Sedum* genus.

	*Sedum* Species					
	*S. sarmentosum*	*S. lineare*	*S. emarginatum*	*S. bulbiferum*	*S. aizoo*	*S. ellacombianum*
	(*n* = 12)	(*n* = 12)	(*n* = 3)	(*n* = 3)	(*n* = 3)	(*n* = 3)
Transferability (%)	100	85.7	78.6	71.4	71.4	64.3

## 3. Experimental Section

### 3.1. Plant Materials and Genomic DNA Extraction

Forty-eight individuals of *S. sarmentosum* were collected from a single population located in Hongshan District, Wuhan, China (30°29′22′′N, 114°23′15′′E). Leaves were randomly sampled and immediately put into the plastic sealed bags with silica gel for fast drying and then stored at room temperature until use. Genomic DNA was extracted from silica-gel-dried leaves using a 2% CTAB protocol with minor modification [30]. The DNA concentration was determined using an ultraviolet-visible spectrophotometer (ND-1000; NanoDrop, Wilmington, DE, USA). The final concentration of each DNA sample was adjusted to 20 ng/µL in TE buffer and kept at −30 °C until use.

### 3.2. Construction of an SSR-Enriched Library and Primer Design

DNA was digested using the restriction enzyme *Mse*I (Promega, Madison, WI, USA) and the digested DNA was ligated to a double-strand linker by T_4_ DNA ligase (TaKaRa, Dalian, China). Then linker-ligated DNA was amplified by 17 cycles of pre-hybridization polymerase chain reaction (PCR) using one strand of the linker as primer. The enriched DNA fragments were denatured and hybridized to biotinylated probes (AC)_15_ followed by incubation with Streptavidin MagneSphere Paramagnetic Particles (Promega, Madison, WI, USA). The captured DNA fragments were used as templates for 33 cycles of PCR amplification using an adaptor as primers. Following amplification, DNA fragments were size-selected (200 to 1000 bp) and purified on agarose gels. The fragments were ligated to pMD18-T vectors and transformed into *Escherichia coli* DH5a competent cells to construct an enriched microsatellite library. Recombinant colonies, identified as white colonies on an LB plate containing ampicillin were screened for inserts using M13 primers and we randomly chose 82 positive colonies to sequence. Tandem Repeats Finder [31] was used to identify the SSR motif and Primer Premier 5.0 software [32] was used to design primers flanking the tandem repeat region in the sequence.

### 3.3. Amplification of SSR, Polymorphism Detection and Data Analysis

The M13-tailed primer method, in which the M13 sequence is attached to the 5′-end of the forward primer, was used to determine the sizes of the amplified products [33]. Microsatellite amplification conditions consisted of 20 µL PCR reaction volumes, which containing 50 ng of genomic DNA, 0.2 mM of each dNTP, 1X Taq polymerase buffer (containing 1.5 mM MgCl_2_), 2 pmol of M13-tailed-forward primer, 4 pmol of the fluorescently labeled M13 universal primer (5′-TGT AAA ACG GCC AGT-3′), 6 pmol of a normal reverse primer, and 1 U of DNA Polymerase (Takara, Dalian, China). The temperature profile was as follows: 5 min initial denaturation at 95 °C, followed by 34 cycles of denaturation at 94 °C for 30 s, annealing at 51–57 °C for 30 s, extension at 72 °C for 1 min and the final extension step of 7 min at 72 °C. The PCR products were electrophoresed on an ABI 3730 genetic analyzer and allele sizes were determined using GeneMapper v4.0 software (Applied Biosystems). The number of alleles per locus (A) and observed (*H*_O_) and expected (*H*_E_) levels of heterozygosity were calculated using the Arlequin program Version 3.5 [34]. Hardy-Weinberg Equilibrium (HWE) and linkage disequilibrium (LD) was tested using the GENEPOP program Version 4.0 [35].

### 3.4. Detection of the Transferability of SSR Primers and Date Analysis

Cross-species amplification tests were performed in randomly selected *S. lineare* (*n* = 12), *S. emarginatum* (*n* = 3), *S. bulbiferum* (*n* = 3), *S. aizoo* (*n* = 3), and *S. ellacombianum* (*n* = 3). The PCR conditions of each loci and the calculation of the genetic diversity parameters were the same as described earlier.

## 4. Conclusions

This is the first set of microsatellite markers developed for *S. sarmentosum*, an important Chinese medicinal herb. A total of 14 polymorphic SSR markers were ultimately screened and proved in *S. sarmentosum*. The SSR primer pairs displayed a high amplification frequency across *Sedum* species. These markers will be valuable for studying the genetic variation, population structure and germplasm characterization of *S. sarmentosum* and related *Sedum* species and aid in the development of conservation practices for *S. sarmentosum*.

## Supplementary Materials

Supplementary materials can be accessed at: http://www.mdpi.com/1420-3049/20/11/19669/s1.
