# Supplementary material for: Development and Characterization of Polymorphic Microsatellite Markers for Sedum sarmentosum (Crassulaceae) and Their Cross-Species Transferability
Source: Molecules. 2015 Nov 5;20(11):19929–35. doi: 10.3390/molecules201119669 (PMC6332276; doi:10.3390/molecules201119669)
Supplement: Supplementary file 1 [file molecules-20-19669-s001.pdf]

*molecules*

# Supplementary Informations

**Supplementary Table.** Transferability of 14 *Sedum sarmentosum* microsatellite markers in other *Sedum* species.

| Locus | *Sedum lineare* | *S. emarginatum* | *S. bulbiferum* | *S. aizoo* | *S. ellacombianum* |
|---|---|---|---|---|---|
| Ssa 47 | – | + | – | – | + |
| Ssa 30 | + | – | – | + | – |
| Ssa 92 | + | + | + | + | + |
| Ssa 46 | + | + | + | + | + |
| Ssa 64 | + | + | – | – | – |
| Ssa 17 | + | + | + | + | + |
| Ssa 66B | + | – | + | + | – |
| Ssa 56 | + | + | + | + | + |
| Ssa 32 | + | + | + | – | – |
| Ssa 10 | + | + | + | + | + |
| Ssa 66A | – | – | – | – | – |
| Ssa 83 | + | + | + | + | + |
| Ssa 60 | + | + | + | + | + |
| Ssa 90 | + | + | + | + | + |
| Transferability (%) | 85.7 | 78.6 | 71.4 | 71.4 | 64.3 |
